# An evidence-based study on the current status of Chinese secondary school mathematics teachers’ autonomous learning capacity across demographic and contextual factors

**DOI:** 10.3389/fpsyg.2022.1042838

**Published:** 2022-10-28

**Authors:** Guangming Wang, Yueyuan Kang, Fengxian Li, Yiming Zhen, Xia Chen, Huixuan Huang

**Affiliations:** ^1^Faculty of Education, Tianjin Normal University, Tianjin, China; ^2^Department of Education, Inner Mongolia Honder University of Arts and Science, Hohhot, China

**Keywords:** mathematics teachers, Chinese teachers, autonomous learning, autonomous learning ability, professional development, teacher learning

## Abstract

Autonomous learning capacity is a key competency that supports teachers’ professional development. In this study, a stratified sampling method was used to recruit 396 junior and senior high school mathematics teachers in T city, one of the provincial city in China. A questionnaire with high reliability and validity developed prior to the study by the researchers was employed to measure their autonomous learning capacity and differences across groups. Twelve teachers were then selected for interviews. The results showed satisfactory overall performance. By subdimension, teachers’ performance was the best in the development of study plans, followed by evaluation of learning outcomes, while they needed improvement in learning habit formation and proficiency in using learning methods. Furthermore, the analysis of differences across groups indicated that for autonomous learning capacity, female teachers were significantly better than their male peers; junior high school teachers were better compared to those in senior high school; teachers aged 41–50 underperformed those aged 51 and above; teachers who work in rural areas and townships did not perform worse than urban teachers; and those with doctoral degrees did not demonstrate considerable advantage over others. There were no significant differences in the overall autonomous learning capacity across years of teaching and job title groups. However, in the subdimensions, those with 21–30 years of teaching experience had lower proficiency in using learning methods and evaluation of learning outcomes, and teachers with senior titles did not demonstrate expected advantages in learning habit formation.

## Introduction

With the demand for highly qualified talent being stronger than ever before, people have become more dependent on learning for personal development. Some believe that “learning” can be autonomous. According to Holec (1979), autonomous learners are those who take responsibility for various decisions throughout the learning process. Individuals cannot learn and develop without external intervention and guidance, including the education provided by teachers. In assessing the professional competencies of teachers, autonomous learning capacity is one of the key indicators (Kang et al., 2021). As learners, teachers need to regard the development of autonomous learning capacity as a lifelong, continuous, and dynamic process (Smith, 2003), forming a mutually reinforcing positive cycle with lifelong learning (Yurdakul, 2017).

Such capacity is important because of two reasons. For students’ learning outcomes, the autonomous learning of teachers contributes to their professional development (Olivier and Wittmann, 2019), and a high level of professional competency is associated with quality teaching (Harris and Sass, 2011; Creemers et al., 2012), which is a predictor of students’ academic achievements (e.g., Sanders et al., 1997; Kunter et al., 2013; Kyriakides et al., 2013). For teachers’ career prospects, autonomous learning capacity and teaching practices are mutually reinforcing. In other words, autonomous learning capacity is both the enabler for and outcome of successful teaching practices. For example, teachers who teach effectively tend to have a higher level of self-efficacy (Holzberger et al., 2013), which motivates them to strive for further professional development and self-improvement (Posnanski, 2002). Meanwhile, quality teaching is often associated with teachers’ characteristics such as personality and educational background (Bowles et al., 2014). Moreover, Certo and Fox (2002) argued that teacher autonomy has a positive impact on perceptions of and attitudes toward teaching. Autonomous teachers are more dedicated to their jobs.

However, studies on mathematics teachers’ autonomous learning capacity, especially on secondary school mathematics teachers, have not been given adequate attention. To a certain extent, assessing teachers’ competencies is also needed to answer the question of why Chinese students can excel consistently in international mathematics tests, such as the Program for International Student Assessment. Research efforts on Chinese mathematics teachers can initiate strategies to improve teacher competencies. Therefore, this study focused on the autonomous learning capacity of secondary school mathematics teachers in China to assess their competencies and analyze their differences by teacher group.

## Literature review

### Teachers’ autonomous learning capacity

Initiative is a key characteristic of autonomous learning (Stockdale and Brockett, 2011). Autonomous learning is the ability to take control of learning (Holec, 1979). Autonomous learners need to proactively evaluate their learning needs, set learning goals, select and implement appropriate learning strategies by leveraging humanistic and material learning resources, and assess learning outcomes with external assistance or under self-motivation (Loeng, 2020). Commenting on learning autonomy, Mynard and Sorflaten (2006) stated that autonomous learners are often confident. They know their strengths and weaknesses, autonomously make learning decisions, pace learning according to actual situations, plan and set their goals, and assess their learning process and progress. Similarly, Oxford (2003) indicated that autonomous learners are highly motivated and self-efficacious and these internal and external motivations can play an active role in helping them achieve better learning outcomes.

For teachers as autonomous learners, in addition to the general characteristics, there are some teacher-specific attributes. First, teachers’ autonomous learning capacity is based on experiences, implying that autonomous learning teachers are proactive, autonomous, and self-directed. Their learning is practice- and problem-oriented and aims at maintaining self-esteem and satisfying needs. In the process of autonomously choosing and integrating new and old knowledge, these teachers fully demonstrate their values (Goodlad, 1990). Moreover, autonomy is a necessary condition for teachers to be creative (Anderson, 2002). Second, teachers’ autonomous learning capacity is derived from addressing questions, suggesting that autonomous learning teachers are reflective and creative. From this perspective, Billett (2002) proposed that teacher learning is a process by which teachers develop skills and acquire knowledge and expertise through reflection and action and that self-regulation of a reflective nature contributes to the development of their autonomy (Papamitsiou and Economides, 2019). Finally, teachers’ autonomous learning capacity is a part of their daily professional competencies. For example, Kelly (2006) defined teacher learning as the process by which teachers aim to gain expertise and that the process of autonomous learning continues to stimulate lifelong learning (Yurdakul, 2017).

In summary, teachers’ autonomous learning capacity involves having autonomy; making plans; taking initiatives; serving professional development needs; and going through the process of autonomous planning, evaluation, and improvement. Accordingly, in this study, teachers’ autonomous learning capacity is defined as teachers’ capabilities to develop and implement their study plans; adopt appropriate means and methods; make full use of time, space, and other resources; and participate in professional activities such as teacher education, educational teaching, and pedagogical research for continuously improving their knowledge and teaching skills.

Smith and Erdogan (2008) explained that such capabilities can be classified into two dimensions: self-directed development and freedom from control. Pintrich (2004) defined autonomous learning as a proactive and constructive learning process and divided the process into three parts: goal setting and planning, execution and action adjustment, and reflection and cognitive monitoring. It is evident that autonomous learning is a means to tackle shortcomings and gain insights about teaching through reflection. From a different perspective Hai and Liu (2018), proposed three analytical dimensions of teacher autonomous learning based on the social cognitive theory: learning motivation, learning strategies and self-monitoring, and argued that teachers’ autonomous learning is a process of professional development based on daily work situations and existing knowledge and experience, in which they consciously and actively use various effective methods and self-regulate to ultimately improve their professionalism and effectiveness. In contrast, Borko (2004) proposed three analytical perspectives on teacher learning based on the contextual learning theory, combined with the views about individual and social cognition. First, the focus is on individual teachers, including their learning activities and mental changes. Second, the focus is on teacher groups, or the communities of practice they belong to and are embedded in during the course of their learning activities. Third, both individual teachers and their groups are considered to examine the impact of individual-environment interactions on teacher learning. From the perspectives of learning behaviors and teacher-specific attributes, teacher learning can be defined as a proactive, autonomous, and self-directed learning process while participating in teacher education, educational teaching, and pedagogical research for professional development.

Sebotsa et al. (2019) suggested that teachers’ professional development may be difficult to sustain without autonomous learning. They recommended including the dimension of autonomous learning to assess teachers’ capabilities in identifying learning needs, setting learning goals, using humanistic and material resources for learning, selecting and implementing appropriate learning strategies, and assessing learning. A similar concern was shared in a discussion about teacher professional development. For example, the National Social Science Fund of China’s 2017 Key Educational Bidding Project “Research on Teachers’ Key Literacy and Competencies” constructed a double-helix model of teachers’ key literacy and competencies and developed a questionnaire based on this model (Wang et al., 2019; Kang et al., 2021). It included teachers’ autonomous learning capacity as one of the competencies and measured it in four subdimensions: development of study plans, proficiency in using learning methods, learning habit formation, and evaluation of learning outcomes. Each subdimension was accompanied by a description of key behavioral requirements for clarity (see Table 1). These subdimensions comprehensively measure competencies mostly related to teachers’ professional capacity, covering the whole process of instructional preparation, implementation, and evaluation. Additionally, the questionnaire was developed in the context of China, where this study was conducted. Therefore, these subdimensions were used in this study.

**Table 1:** Subdimensions of teachers’ autonomous learning capacity and behavioral requirements.

	Subdimensions	Key behavioral requirements
Teachers’ autonomous learning capacity	Development of study plans	Teachers are able to understand the requirements of professional standards, undergo self-reflection, and adopt suggestions from colleagues, thus making study plans that address their professional learning needs and setting clear learning goals for the expected outcomes of their professional learning.
	Proficiency in using learning methods	Teachers are able to integrate the following learning activities: reading books and journals, browsing professional websites, observing public classes, watching videos of quality classes, attending special lectures, taking online courses, and taking learning trips.
	Learning habit formation	Teachers are able to develop the habit of taking initiative in learning and form the habit of lifelong learning over time.
	Evaluation of learning outcomes	Teachers are able to evaluate their learning process comprehensively through self-evaluation, learning summaries, scales, and surveys, covering the measures of the initiative and continuity of learning, the effectiveness in applying learning methods and strategies, problems that arise in the learning process, and learning outcomes.

### Impact of demographic and contextual factors on teachers’ autonomous learning capacity

Learning is influenced by three types of factors: personal factors, environmental factors, and behaviors. These factors affect each other reciprocally in a continuous cycle (Bandura, 1962). Among them, the environment can be social or physical, and thus, classified under external factors; while personal factors and behaviors can be categorized under internal factors. For external factors, teacher learning and development take place in professional communities (Shulman and Shulman, 2009; De Jong et al., 2019), and an empirical study revealed that a school’s learning beliefs, learning support systems, and learning communities impact teachers’ professional learning (Opfer et al., 2011). Moreover, a study of primary and secondary school teachers’ motivation for professional development found that external contextual factors such as educational stage, location, and years of teaching experience have significant effects on teachers’ motivation for learning and development (Qi et al., 2020). In addition, in a longitudinal study, Day and Gu (2007) found that given the humanistic aspects of teachers’ work, not only the external factors across different stages of professional development but also internal conditions can affect their professional learning. For example, personal changes and changes in a work environment can have different effects on professional learning at different stages of career development.

Regarding internal factors, teacher competencies have been graded from the perspective of individual knowledge building (Shulman, 2011) or an individual’s positioning within a community (De Jong et al., 2019). Subsequent studies have addressed differences at the individual level. For example, Abdel Razeq (2014) found significant differences in teachers’ autonomous learning capacity by gender, with female teachers being better at autonomous learning. Deregözü and Hatipoğlu (2018) identified no differences in German pre-service teachers’ autonomous learning capacity by grade level and educational background but revealed statistical differences between them by age and gender. Using data from the 2013 Teaching and Learning International Survey, Chen (2017) added the factor of years of teaching experience and empirically examined teachers’ professional development in 36 jurisdictions. The results showed that contextual factors, such as teachers’ gender, years of teaching experience, educational background, and professional development needs, have significant effects on teachers’ professional learning and development. Besides, a survey of kindergarten teachers in China conducted by Hai and Liu (2018) found significant differences in autonomous learning among teachers with different years of teaching experience, educational background, professional backgrounds, and job titles. The autonomous learning of teachers is determined to a larger extent by individual internal factors than by external factors such as school and school location.

Summarily, teachers’ autonomous learning capacity is affected by a variety of internal and external conditions, including gender, age, years of teaching experience, educational background, job title, educational stage, and location. However, the majority of these studies have failed to take into consideration teachers’ educational stages or the subject they teach, let alone focus on secondary school mathematics teachers. Moreover, the resultant recommendations for improvement are mostly generic, rather than specific enough for a teacher to incorporate them in their teaching practices for a particular subject. To address the issue, personal factors and external school factors identified in prior studies on differences among teacher groups can be repurposed as demographic and contextual factors. Therefore, this study aimed to examine the following two research questions:

RQ1: What is the overall status of the autonomous learning capacity of secondary school mathematics teachers?

RQ2: Are there differences in autonomous learning capacity among teacher groups across the contextual factors of gender, age, years of teaching experience, educational background, job title, educational stage, and location? If yes, what are the differences?

## Materials and methods

### Participants

T city is a provincial city in northern China with good education and economic development. To balance the level of teachers in different schools and to enhance the representativeness of the sample, a stratified sampling method was used to select 430 secondary school mathematics teachers from 8 out of 16 districts of T city as anonymous survey respondents and face-to-face questionnaire completion. Among them, 73 were males and 319 were females. They were aged between 31 and 50 years old, with largely 11–30 years of teaching experience. A majority of them have completed undergraduate education. Most of them work in urban areas. Most of the job titles were intermediate and senior ones (see Table 2 for detailed information about the respondents). Among them, the job title is the result of the evaluation of the level of primary and secondary school teachers by the regional education bureaus in China according to the Evaluation Standards for the Professional and Technical Levels of Primary and Secondary School Teachers in China. In the test, we divided the job titles into three categories, namely not rated or primary, intermediate and senior, according to the level of teachers in T city. A total of 430 questionnaires were distributed. Using polygraph questions, 34 invalid questionnaires with inconsistent responses in the teacher autonomy questionnaire (the second part of the questionnaire) were eliminated, 396 valid questionnaires were obtained, with a return rate of 92.09%.

**Table 2:** Basic information of teachers surveyed.

Demographic variables	Categories	*N*	Percentage (%)
Gender	Male	73	18.39
	Female	319	80.35
	Not available	4	1.26
Age (in years)	30 and below	55	13.89
	31–40	123	31.06
	41–50	153	38.64
	51 and above	64	16.16
	Not available	1	0.25
Years of teaching experience	10 and below	81	20.45
	11–20	143	36.11
	21–30	128	32.32
	31 and above	41	10.35
	Not available	3	0.76
Education	Associate and below	9	2.27
	Bachelor	298	75.25
	Master	83	20.96
	Doctor	5	1.26
	Not available	1	0.25
Job title	Not rated or primary	47	11.87
	Intermediate	183	46.21
	Senior	165	41.67
	Not available	1	0.25
Educational stage	Junior high school	243	61.36
	Senior high school	153	38.64
Location	Urban area	243	61.36
	Township	123	31.06
	Rural area	21	5.30
	Not available	9	2.27

“Not available” indicates an invalid response by the teacher in the first part of the questionnaire for background information.

For a more robust interpretation of the survey results, purposive sampling was used, thus providing more information for this study (Chen, 2000). We selected 12 mathematics teachers and conducted semi-structured interviews using a combination of individual interviews, focus group interviews, phone calls, and online interviews. Moreover, the purposive sampling covered teachers from different groups to ensure representativeness. Specifically, among the 12 mathematics teachers, some were from junior high schools, while others were from senior high schools, and other teacher groups were also well-represented. Among them, 33.34% were males and 66.66% were females; 25% were aged 30 years and below, 41.67% were aged 31–40 years, and 33.33% were aged 41–50 years; 41.67% had taught for 10 years and below, 33.33% for 11–20 years, and 25% for 21–30 years. Regarding job titles, 25% were at junior level and below, 33.33% were at an intermediate level, and 41.67% were at senior level. In the survey process, first, researchers set up a survey team and trained the members to ensure that they fully grasped the purpose and method of the survey; then, with the consent of the teachers, we introduced the purpose, method and data analysis results of the study to the teachers; finally, we interviewed the teachers according to the interview questionnaire, using shorthand text recording, respecting the interviewees’ request not to record their voices, and keeping the interview text strictly confidential and anonymous to fully protect the privacy of the interviewees.

### Instruments

The questionnaire used in this study was answered anonymously and consisted of two parts—teachers’ basic information and their autonomous learning capacity— with a total of 18 questions. The first part covered the teachers’ personal factors and external contextual factors (gender, age, years of teaching experience, educational background, job title, educational stage, and location). The second part was the Teachers’ Autonomous Learning Capacity Scale. The questions were mostly drawn from the teachers’ autonomous learning capacity items of the Teachers’ Key Literacy and Competencies Scale devised by Kang et al. (2021) and structured according to the scale of Williamson (2007). The final version of the questionnaire comprised four subdimensions: development of study plans, proficiency in using learning methods, learning habit formation, and evaluation of learning outcomes. To avoid subjective judgment and biased results related to the “do you agree” questions, experience, means, or situation-based questions were used, mostly about the teachers’ behaviors or performance in teacher education, educational teaching, and pedagogical research activities. For example, a question about the development of study plans reads: Which of the following statements best describes your experience in the development of study plans in the recent 5 years? A. Have never made a personal study plan. B. Have included specific study plans in work plans for 1–3 semesters. C. Have included specific study plans in work plans for 4–6 semesters. D. Have included specific study plans in work plans for seven or more semesters.

Using the SPSS 24.0, the reliability test of the scale determined using Cronbach’s *a* yielded a value of 0.825, indicating high reliability. Correlation analysis was performed to examine the scale’s structural validity. The correlation coefficients between the subdimensions ranged from 0.36 to 0.60, implying a medium correlation between the indicators. The correlation coefficients between the subdimensions and the overall measure ranged from 0.62 to 0.91, indicating a high correlation between the four subdimensions and teachers’ autonomous learning capacity (see Table 3), and overall good structural validity of the scale.

**Table 3:** Correlation between teachers’ autonomous learning capacity and its subdimensions.

	Autonomous learning capacity	Development of study plans	Proficiency in using learning methods	Learning habit formation	Evaluation of learning outcomes
Development of study plans	0.768^**^	1			
Proficiency in using learning methods	0.622^**^	0.368^**^	1		
Learning habit formation	0.762^**^	0.452^**^	0.454^**^	1	
Evaluation of learning outcomes	0.903^**^	0.592^**^	0.420^**^	0.536^**^	1

** *p* < 0.01.

The interview outline included four themes: development of study plans, proficiency in using learning methods, learning habit formation, and evaluation of learning outcomes; these can be mapped to the four subdimensions of the scale. To gain a deeper understanding of the interviewers’ experience with autonomous learning and its influencing factors, six items were developed based on the outline. Then, they were revised again to incorporate inputs from experts after the interview instrument was drafted. The questions are as follows: (1) Do you make a study plan before autonomous learning? How long is the time horizon covered in the study plan? What are the factors that influence your decision for developing a study plan? (2) Do you use anything as a reference when making a study plan? As a mathematics teacher, what are the areas involved in autonomous learning? (3) In what way do you carry out autonomous learning? What are the factors that influence your decision on choosing the learning methods? (4) How long is the cycle of your autonomous learning? What steps do you usually take to form a learning habit? What do you think are the reasons why “there is no time for autonomous learning?” (5) Do you assess your own learning? What areas are considered for the assessment? What are the factors that influence your decision for assessing your learning? (6) What should be done to improve teachers’ autonomous learning capacity? What are your expected supports from your family, school, and community?

### Data analysis

Addressing research question 1, SPSS 24.0 was used for descriptive statistical analysis of the data and for calculating score rates of teachers’ autonomous learning capacity and the subdimensions under it. The results presented an overall picture of the autonomous learning capacity of secondary school mathematics teachers. The score rates were calculated as the percentage ratios of the scores to the total points (score rate = score/total points for a dimension). Higher score rates indicate higher levels of teacher capacity in that dimension.

For research question 2, *t*-tests and one-way analysis of variance (ANOVA) were used to test for differences in teachers’ autonomous learning capacity across gender, age, years of teaching experience, educational background, job title, location, and educational stage groups. For groups with significant differences, the least significant difference test was used *post hoc* to identify specific differences between the teachers. Furthermore, the effect sizes were calculated to analyze the differences. Specifically, *η*^2^ = 0.01 (or *d* = 0.2), *η*^2^ = 0.06 (or *d* = 0.5), and *η*^2^ = 0.14 (or *d* = 0.8) were used to represent small, medium, and large effects, respectively (Cohen, 1988). Finally, for a robust interpretation of the survey results and a more comprehensive understanding of the factors affecting teachers’ autonomous learning capacity, the interview outline was used for the teachers’ semi-structured interviews. Two researchers conducted independent qualitative analyses of the interview transcript to summarize the teachers’ main views using a bottom-up approach.

## Results

### Current autonomous learning capacity of secondary school mathematics teachers

Regarding the overall results of the autonomous learning capacity of secondary school mathematics teachers, the mean score was 29.31 with a score rate of 81.42%. The score rate can also be interpreted as the percentage score, which indicates that the overall level of teachers’ autonomous learning ability, measured on a percentage scale, reached a corresponding level of 81.42 points, which is a good level. The highest score of 36 had a high frequency, indicating that there were multiple outstanding teachers. The lowest score of 16 had a low frequency, suggesting a desirable result.

**Figure 1:**
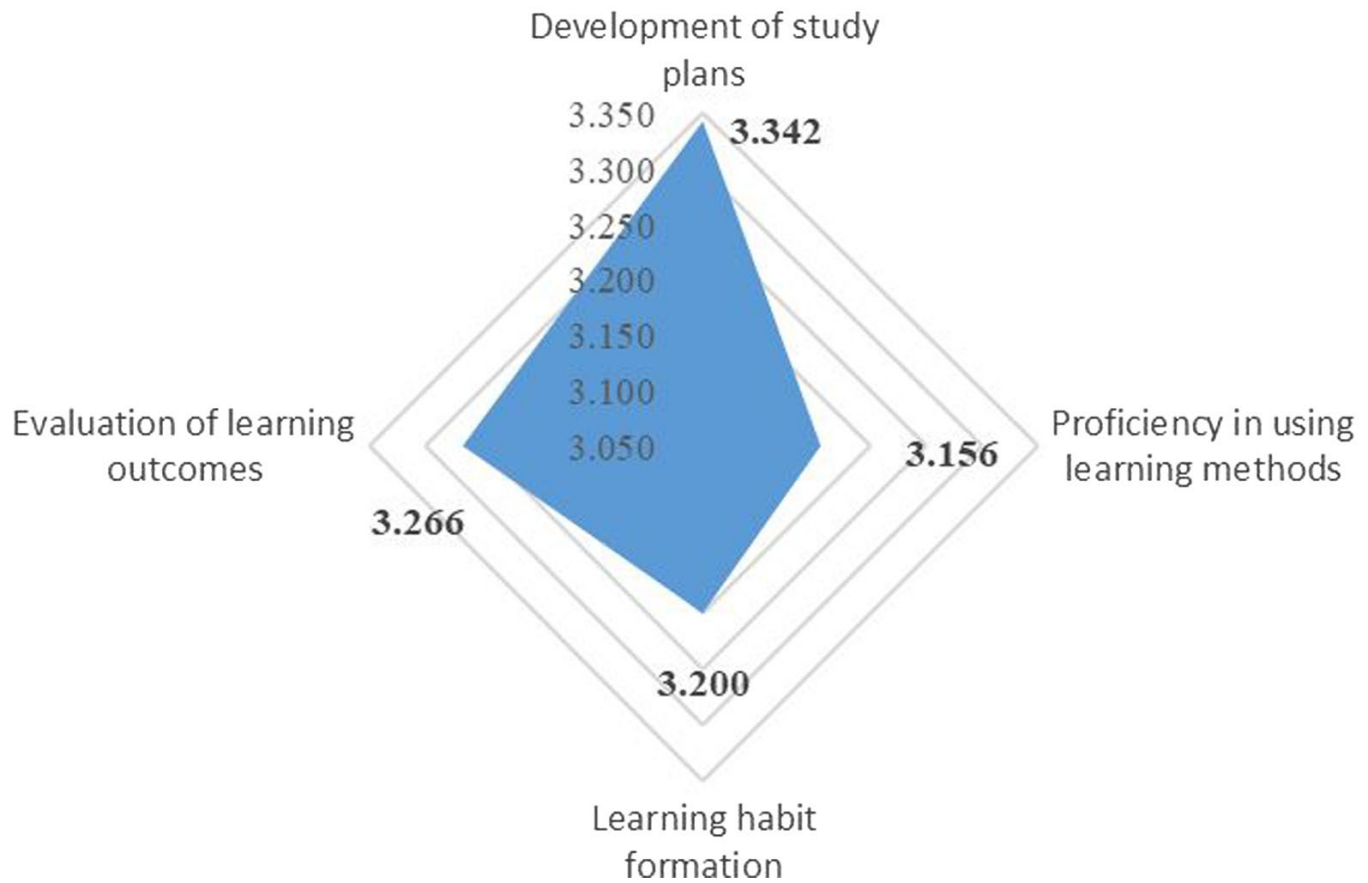
Subdimension means of teachers’ autonomous learning capacity.

By subdimension (see Table 4 for detailed results), the results of mean and median scores greater than 3, negative kurtosis and skewness, and the left-skewed distribution indicated that the scores are generally high and most teachers have good performance. To be specific, teachers had the best performance in the development of study plans (*M* = 3.342), satisfactory performance in the evaluation of learning outcomes (*M* = 3.266), and poor performance in learning habit formation (*M* = 3.200) and proficiency in using learning methods (*M* = 3.156), as shown in Figure 1.

**Table 4:** Descriptive results of teachers’ autonomous learning capacity by subdimension.

	*N*	Mean (*M*)	Median	Standard deviation	Variance	Skewness	Kurtosis	Min.	Max.
Development of study plans	397	3.342	3.500	0.677	0.458	−0.758	−0.284	1.00	4.00
Proficiency in using learning methods	397	3.156	3.000	0.894	0.799	−0.802	−0.224	1.00	4.00
Learning habit formation	397	3.200	3.500	0.708	0.501	−0.713	−0.108	1.00	4.00
Evaluation of learning outcomes	397	3.266	3.250	0.653	0.426	−0.549	−0.669	1.25	4.00

Regarding particular items, in the development of study plans, 97.7% of teachers included specific study plans in their work plans for varying lengths (in semesters), while 2.3% of teachers had never made a personal study plan, indicating that most teachers perform well in this subdimension. In the proficiency in using learning methods, we primarily examined whether teachers were able to conduct autonomous learning in combination with the following methods: reading books or journals, browsing professional websites, observing public classes, watching videos of quality classes, attending lectures, participating in online courses, and visiting field trips. Moreover, we found that 78.3% of teachers were aware of all the above learning methods and used at least half of them in learning, 15.9% of teachers were aware of half of them and used a few for effective learning, and 5.8% of teachers did not know the learning methods and had never used them, indicating a good performance by most teachers in this subdimension. In the learning habit formation, 73.2% of teachers reported taking some time to study regardless of whether they are busy at work or not, 22.5% only studied intermittently, and 4.3% had no time to study, indicating that while most teachers have an established study habit, there is still room for further improvement. In the evaluation of learning outcomes, we mainly examined whether teachers can make a comprehensive assessment of the autonomous learning process, including the initiative of learning, continuity of learning, effectiveness of learning methods, effectiveness of learning strategies, and smoothness of the learning process. It was found that 92.9% of teachers could diagnose at least two of the above aspects, and only 7.1% of teachers did not evaluate any aspect of the learning process, indicating that most teachers perform well in this subdimension.

### Analysis of differences across teacher groups

To examine the effects of different factors on teachers’ autonomous learning capacity, independent samples t-tests were conducted for gender and educational stage. The results showed significant differences with small to medium effect sizes in autonomous learning capacity between male and female mathematics teachers (*t* = −2.067, *p* = 0.039 < 0.05, *d* = 0.259), and between junior and senior high school teachers (*t* = 2.796, *p* = 0.006 < 0.01, *d* = 0.292). Specifically, female teachers outperformed male teachers, and junior high school teachers outperformed senior high school teachers.

Then, one-way ANOVA tests were conducted for age, years of teaching experience, educational background, job title, and locational factors. Significant differences with small to medium effect sizes were identified for teachers in age (*F* = 0.045, *p* = 0.045 < 0.05, *η^2^* = 0.020), educational background (*F* = 2.650, *p* = 0.049 < 0.05, *η^2^* = 0.020), and location (*F* = 6.575, *p* = 0.002 < 0.01, *η^2^* = 0.033). Specifically, teachers aged 51 years and above performed significantly better than those in the 41–50 age group, teachers who worked in rural areas and townships were significantly better than urban teachers, and teachers with master’s degrees and below were significantly better than those with doctoral degrees in autonomous learning capacity (see Table 5). In addition, there was no significant difference in the autonomous learning capacity of mathematics teachers in terms of years of teaching and job title (*p* > 0.05).

**Table 5:** One-way analysis of variance for teachers’ autonomous learning capacity.

	Categories		*N*	Mean	SD	*F*	*Post-hoc* tests	*η*²
Age	A	30 and below	55	30.073	5.319	2.713^*^	C < D	0.020
	B	31–40	123	29.252	4.970			
	C	41–50	153	28.549	5.219			
	D	51 and above	65	30.436	4.1548			
Educational background	A	Associate and below	9	29.223	2.539	2.650^*^	C > D	0.020
	B	Bachelor	298	29.594	4.975			
	C	Master	83	28.615	5.277			
	D	Doctor	6	24.5623	5.4360			
Location	A	Urban areas	244	28.623	4.967	6.575^**^	A < B	0.033
	B	Township	123	30.255	5.095			
	C	Rural area	21	31.476	3.945			

* *p* < 0.05 and

** *p* < 0.01.

To further explore the differences in teachers’ autonomous learning capacity across subdimensions, independent samples t-tests were conducted for each of the four subdimensions with respect to gender and educational stage. The results showed that female teachers were significantly better than male teachers in the evaluation of learning outcomes (*t* = 2.540, *p* = 0.013 < 0.05, *d* = 0.345), and junior high school teachers were significantly better than senior high school teachers in learning habit formation (*t* = 2.078, *p* = 0.038 < 0.05, *d* = 0.213), with small to medium effect sizes.

Then, one-way ANOVA tests were conducted for age, years of teaching experience, educational background, job title, and locational factors successively for each of the four subdimensions (see Table 6). Significant differences with small to medium effect sizes were identified in teachers across age (*F* = 3.757, *p* = 0.011 < 0.05, *η^2^* = 0.028) and location (*F* = 4.084, *p* = 0.018 < 0.05, *η^2^* = 0.021) in development of study plans. Specifically, teachers aged 51 years and above significantly outperformed teachers aged 31–50 years, and rural teachers outperformed urban teachers.

**Table 6:** One-way analysis of variance for teachers’ autonomous learning capacity by subdimension.

Subdimensions	Categories		*N*	*Mean*	*SD*	*F*	*Post hoc* tests	*η^2^*
Development of study plans	Age (in years)	A. 30 and below	55	3.309	0.773	3.757*	C < D	0.028
		B. 31–40	123	3.333	0.706			
		C. 41–50	153	3.255	0.667			
		D. 51 and above	65	3.585	0.489			
	Location	A. Urban areas	244	3.271	0.693	4.084*	A < C	0.021
		B. Township	123	3.438	0.667			
		C. Rural area	21	3.595	0.436			
Proficiency in using learning methods	Age (in years)	A. 30 and below	55	3.182	0.819	3.468*	B > C	0.026
		B. 31–40	123	3.309	0.780			
		C. 41–50	153	2.981	0.970			
		D. 51 and above	65	3.246	0.919			
	Years of teaching experience	A. 10 and below	81	3.222	0.791	3.213*	B > C	0.024
		B. 11–20	143	3.308	0.824			
		C. 21–30	129	2.985	0.984			
		D. 31 and above	41	3.098	0.944	4.523*	A < C	0.023
	Location	A. Urban areas	244	3.074	0.944			
		B. Township	123	3.244	0.803			
		C. Rural area	21	3.619	0.590			
Learning habit formation	Educational background	A. Associate and below	9	3.333	0.500	5.958**	B > C A > D B > D C > D	0.044
		B. Bachelor	298	3.251	0.672			
		C. Master	83	3.072	0.770			
		D. Doctor	6	2.170	0.982			
	Job title	A. Junior and below	47	3.340	0.635	2.862*	B > C	0.021
		IB. ntermediate	183	3.154	0.701			
		C. Senior	165	3.215	0.723			
Evaluation of learning outcomes	Years of teaching experience	A. 10 and below	81	3.414	0.64488	3.015*	C < D	0.023
		B. 11–20	143	3.242	0.62810			
		C. 21–30	129	3.173	0.68982			
		D. 31 and above	41	3.404	0.51782			
	Location	A. Urban areas	244	3.181	0.66001	6.029**	A < B	0.030
		B. Township	123	3.393	0.61987			
		C. Rural area	21	3.512	0.60455			

* *p* < 0.05 and

** *p* < 0.01.

Significant differences with small to medium effect sizes were identified in teachers across age (*F* = 3.468, *p* = 0.016 < 0.05, *η^2^* = 0.026), years of teaching experience (*F* = 3.213, *p* = 0.023 < 0.05, *η^2^* = 0.024), and location (*F* = 4.523, *p* = 0.011 < 0.05, *η^2^* = 0.023) in proficiency in using learning methods. Specifically, teachers aged 41–50 years were less proficient than those aged 31–40 years and over 51 years.

Significant differences with small to medium effect sizes were identified in teachers across educational background (*F* = 5.958, *p* = 0.001 < 0.01, *η^2^* = 0.044) and job title (*F* = 2.862, *p* = 0.037 < 0.05, *η^2^* = 0.021) in learning habit formation. Specifically, teachers with master’s degrees and below performed significantly better than those with doctoral degrees, teachers with bachelor’s degrees performed significantly better than those with master’s degrees, and teachers with senior titles significantly underperformed those with intermediate and below titles.

Significant differences with small to medium effect sizes were identified in teachers across years of teaching experience (*F* = 3.015, *p* = 0.030 < 0.05, *η^2^* = 0.023) and location (*F* = 6.029, *p* = 0.003 < 0.01, *η^2^* = 0.030) in evaluation of learning outcomes. Specifically, teachers with 21–30 years of teaching experience significantly underperformed teachers with 10 years and below and 31 years and more of teaching experience, and township teachers performed significantly better than urban teachers.

### Qualitative analysis of interview data

Semi-structured interviews were conducted with 12 middle and high school mathematics teachers to assist in interpreting the results of the data analysis. The qualitative analysis revealed that the differences in autonomous learning capacity across groups could be explained by both internal and external factors.

#### Internal factors

##### Age and years of teaching experience: Mathematics knowledge base

During the interviews, most teachers agreed that adequate mathematics expertise could increase their efficiency in autonomous learning and acquiring new knowledge and skills. The perception of improved self-efficacy, in turn, could initiate a virtuous cycle for continuous autonomous learning. The majority of the participants explained that teachers with domain knowledge of mathematics are more likely to understand what they are learning because they can relate new knowledge with what they already know. The network of knowledge means that they better locate anything within the knowledge system, which provides them with a holistic view to approach new knowledge, thus achieving better learning efficiency.

For example, Teacher A said: “I feel that the obvious gap between myself and veteran teachers is that my mathematics-related knowledge (for example, history of mathematics, mathematical analysis, calculation skills) is not as extensive as theirs. I spend much more time than they spend in acquiring the same new knowledge. Also, they are more thoughtful. I often miss the points that veteran teachers may raise. Besides, my views on some of the frontier issues can also enlighten veteran teachers.”

Teacher E said: “In a workshop on what ‘differences’ should be reflected in the approach of ‘adopting different methods for the same mathematics lesson,’ I thought of a teacher’s personal teaching style, handling of teaching content, teaching ideas and methods, and the use of teaching tools. However, a veteran teacher mentioned the importance of paying attention to the students’ differences when planning a ‘different’ lesson. This is something I had not thought of.”

The results of the above interviews reveal that while newly recruited young teachers may be inexperienced, they are more capable and motivated for self-reflection and autonomous learning, and are more concerned with the frontier of the subject. Veteran teachers, on the other hand, have a greater advantage in terms of their knowledge base in mathematics.

##### Location: Learning methods

The interviews suggested that appropriate learning methods contribute to mathematics teachers’ efficient learning. The rapid development of technologies, including multimedia technology and artificial intelligence, has expanded available applications for mathematics and methods for autonomous learning. As a provincial and a coastal port city in China, T city has few agricultural areas and mountainous areas, a better level of education informatization, and a wide coverage of teacher training and post-service training power, so teachers in rural areas have access to rich learning resources and opportunities. The right approach can effectively improve the efficiency of teachers’ autonomous learning and learning outcomes. Choosing appropriate and suitable learning methods is a necessary prerequisite for teachers to engage in autonomous learning.

For example, Teacher D said: “To improve my professional competencies, I use online resources for autonomous learning, such as watching recorded videos for national high-quality lessons and learning information technology and pedagogical skills. I also participate in the mathematics pedagogical research activities organized by the school to learn from the experiences of other teachers.”

Teacher C said: “Although our school is located in a remote location, thanks to the development of technologies, China has made many databases publicly accessible. I can observe online quality lessons delivered by mathematics teachers nationwide and learn the way excellent teachers handle their classes.”

Teacher E said: “I try to improve my knowledge and professionalism through mathematics teaching reference books. Now that it’s easy to get around, sometimes, I also go to downtown schools to attend lectures by experts in mathematics education to get my questions about learning and teaching answered. In addition, I think lesson preparation is also a good way to learn. I acquire or learn new ideas every time I prepare a lesson.”

##### Gender, educational stage, and educational background: Internal motivations for learning

The interviews revealed that internal motivations are a necessary prerequisite for teachers’ autonomous learning. What motivates teachers to learn autonomously can be internal, including their personal values and goals for self-improvement. These motivations are catalytic and inspirational, encouraging teachers to enhance their professional knowledge and skills, knowledge base, and determination in overcoming difficulties they encounter in the learning process continuously and sustainably.

For example, Teacher F said: “As a female teacher, I am often able to derive satisfaction from teaching. My self-improvement is driven by good interaction with students in the classroom and the improvement in student achievement. I study regularly and autonomously. I find ways to overcome any difficulties and obstacles I encounter in the learning process, rather than giving up.”

Teacher G said: “I teach senior high school mathematics, which is difficult, so I usually spend most of my time teaching and preparing lessons. My main goal is to help students improve their grades in the college entrance examination, so I have little need to study myself and have no set study habit.”

Teacher K said: “As a teacher who has experienced systematic research training at the doctoral level, I believe that my mathematical expertise meets the requirements for teaching, so I do not need much additional independent study. There are few mathematics teachers with doctoral degrees, and the teachers around me often talk to me about professional issues. I am not particularly knowledgeable about curriculum standards and other content, and usually do not spend time studying them.”

From the results of the above interviews, it is evident that female teachers are able to gain satisfaction from teaching mathematics and have a high internal motivation for learning, a strong sense of autonomous learning, and personal learning needs and pursuits. In contrast, senior high school teachers are more dedicated to teaching, preparing lessons, and helping students improve their academic performance in mathematics and prepare for the college entrance examination. They have weak internal motivation for learning and forming learning habits. Additionally, the doctoral-educated teachers who participated in this study had science and technology academic backgrounds, had not experienced teacher education, and had weaker knowledge of educational expertise, which, combined with the security that comes with a deep foundation of mathematical expertise, prevented them from developing the habit of autonomous learning. This finding corroborates and explains the results of the questionnaire survey that “in autonomous learning capacity, female teachers are significantly better than male teachers, junior high school teachers are better than senior high school teachers, and doctoral teachers underperformed.”

#### External factors

##### Age and years of teaching experience: Learning time

In the interviews, most teachers mentioned that they had no free time for autonomous learning due to the numerous chores at home and the heavy workload. With little free time at their disposal, they had time for autonomous learning only during semester breaks, but they still had to prepare lessons for the semester. As a result, many teachers’ autonomous learning covers only knowledge closely related to the subjects they teach.

Teacher H said: “Classroom work, various interpersonal activities, and regular teaching take up most of the time. In addition, as middle-aged people, they have to take care of their families and children. Thus, they rarely have time for autonomous learning and usually study for completing the tasks assigned by the school.”

Teacher B said: “The school conducts a lot of teacher training programs. As middle-aged teachers, we have too many chores in our family life, so we do not have time for autonomous learning.”

##### Job titles and educational background: Reward and evaluation policies

Sound evaluation policies are critical to measure the quality of teachers’ autonomous learning. Schools play a major role in teacher evaluation; therefore, a robust evaluation framework is needed in schools to motivate teachers for autonomous learning. The interviews revealed that teacher evaluation policies place too much emphasis on differentiation and selection. Accordingly, teachers focus on these performance indicators, particularly on students’ academic achievements. They have few incentives for enhancing their professional competencies through autonomous learning.

For example, Teacher L said: “The school’s evaluation policies are based on achievements of students in the class, the papers published by teachers, and the rankings achieved in competitions. Even those who want to learn are targeting promotion or job titles. Teachers are not motivated to learn for improved knowledge and learning capacity.”

Teacher K said: “teachers with a doctoral degree have certain advantages in the job title evaluation, as a doctoral degree mathematics teacher, my professional knowledge is relatively more solid, and I usually like to study and research mathematics knowledge.”

The results of the above interviews identified a lack of time for learning, limited energy, and work and family factors that weaken autonomous learning capacity among middle-aged teachers. In addition, the interviews revealed that teachers with senior job titles and doctoral degrees are not sufficiently incentivized for enhancing autonomous learning capacity by the reward and evaluation policies.

## Discussion

### Satisfactory autonomous learning capacity of secondary school mathematics teachers

Considering the findings of Shao (2013) and Sun (2012), this study revealed generally satisfactory autonomous learning capacity of secondary school mathematics teachers. Specifically, among the four subdimensions, teachers’ ability in the development of study plans was the best, indicating that most teachers can set clear learning goals and develop study plans based on professional requirements through self-reflection, incorporating suggestions from colleagues, and considering their own professional learning needs. However, the interviews revealed that teachers’ planning cycles generally last for a semester. They mostly focus on the next semester’s course schedule rather than on professional self-improvement. Moreover, teachers’ ability to evaluate learning outcomes is satisfactory, indicating that they can use a combination of assessment tools to evaluate learning outcomes. However, it was revealed that some teachers are overly interested in results and have little appetite for reflecting on the learning process. Finally, improvements are needed in teachers’ abilities to form learning habits and gain proficiency in using learning methods, both of which require practice and time. In the interview, we found that although teachers have a desire to learn and improve, many of them report that their study habits are interrupted by external factors due to work commitments and life chores, and they lack time for autonomous learning. In addition, increased job stress can lead to a decrease in their job satisfaction and a lack of time for professional development and enhancement (Naylor, 2001), which can weaken their competency.

### Differences in autonomous learning capacity of secondary school mathematics teachers across contextual factors

The results revealed significant differences in teachers’ autonomous learning capacity across gender, age, years of teaching experience, educational stage, and location. First, between gender groups, female teachers’ autonomous learning capacity is significantly better than that of male teachers, especially in terms of evaluation of learning outcomes. The reason is that female teachers in compulsory educational stages in China have higher job satisfaction than males (Li et al., 2017). As a result, it is easier for female teachers to receive positive feedback in their teaching. Their increased self-efficacy helps them make better career decisions (Ince Aka and Tasar, 2020), motivating them to learn autonomously for professional improvement, which was also verified in the interviews. Self-evaluation of learning outcomes helps them diagnose their shortcomings and find the right direction for learning improvement. In addition, the results of the interviews suggest that teachers’ internal motivation contributes to the improvement of their autonomous learning capacity. These results can also explain the finding.

Among educational stages, junior high school teachers have better levels of autonomous learning capacity, especially in learning habit formation. The interviews also revealed that some senior high school teachers mistakenly equate a higher educational stage with higher competencies, resulting in a low level of enthusiasm for their professional advancement. It was also revealed that teachers’ lack of internal motivation to learn can affect their planning for autonomous learning, making it difficult for them to form study habits. Moreover, a higher level of educational stage is accompanied by increased pressure due to the college entrance examinations that students in these classes undertake. Senior high school teachers devote most of their energy to education and teaching, instead of paying attention to autonomous learning for self-improvement. In addition, the interviews also showed that the utilitarian orientation of reward and evaluation policies, as well as less rewarding teacher training activities, occupy too much of the available time for teachers. The situation is worsened by the fact that teachers’ professional learning and development are mostly driven by external sources (Lefstein et al., 2020). As a result, teachers have no time for autonomous learning and maintaining learning habits.

Among age groups, teachers aged 41–50 years are less capable of autonomous learning than teachers aged 51 years and above, especially in terms of the development of study plans. In contrast, the former are less adept than those aged 31–40 years in proficiency in using learning methods. This finding is in line that of with previous studies (e.g., Huberman, 1989; Liu and Liu, 2019), and is understandable because teachers aged 41–50 years have transformed from novice to skilled teachers. Given the accumulated work experience, they are prone to burnout, leading to an insufficient passion for learning and development. Meanwhile, the finding that school learning is most conducive to teachers’ professional learning and development (Postholm, 2012) implies that middle-aged teachers are in a disadvantaged position for autonomous learning because they need to balance their work and familial responsibilities, which was verified in the interviews. Furthermore, the interviews indicated that teachers’ knowledge base in mathematics also affects their autonomous learning capacity. Teachers aged 41–50 years were less knowledgeable and experienced in mathematics than senior teachers and less refreshed than younger teachers. The burnout effects can further decrease their motivation for autonomous learning.

There were no significant overall differences between teachers based on years of teaching experience. However, in the subdimensions, teachers with 21–30 years of teaching experience had low proficiency in using learning methods compared to those with 11–20 years of teaching experience and poor evaluation of learning outcomes compared to those with 31 years and more teaching experience. A previous study revealed that teachers with 6–10 years of teaching experience have the highest level of career attraction and are in the prime of their career development, while teachers over 40 years of age show negative performance and lack the motivation to learn (Liu and Liu, 2019). Huberman (1989) divided the professional life cycle of teachers into seven stages, in which the period of calm and relational distancing can portray the psychological state of teachers with 21–30 years of teaching experience. Many teachers at this stage begin to calm down after experiencing doubt and crisis and are able to complete their classroom teaching with greater ease and self-confidence. However, as career aspirations are gradually achieved, their level of ambition starts declining (Ye, 2001), and the need for self-evaluation decreases. In addition, the interview results suggested that learning methods and internal learning motivations affect the development of this group of teachers’ learning capacity. Teachers in a period of calm and relational distancing also have a reduced willingness to explore learning methods. This explains part of the differences with other teachers.

Among location groups, rural or township teachers did not show deficiencies in overall autonomous learning capacity or in the development of study plans, proficiency in using learning methods, and evaluation of learning outcomes. This finding differs from those that suggested that rural teachers need to improve their competencies because they are mediocre (Li and Cui, 2015). One possible reason to explain the inconsistency is that collaboration among teachers contributes to their professional learning and development (Smith et al., 2020). Participants of this study were from the areas of Chinese provincial and coastal cities, with few agricultural and mountainous areas, a good level of education informatization, and a wide coverage of teacher training and post-service training efforts. The rural teachers in the interviews also indicated that they had access to abundant learning resources and the opportunity to learn independently using a variety of learning methods and to collaborate with teachers from other regions. Efforts over the years for quality and balanced development of compulsory education and provision of special training for rural teachers have yielded positive result. Additionally, the job stress of rural teachers is relatively low, giving them more time for autonomous learning. To compensate for the lack of resources in townships and rural areas, teachers in these areas have more moral duties for self-improvement, resulting in higher autonomous learning capacity of rural teachers than those of urban peers across all subdimensions.

Among educational background groups, those with doctoral degrees did not have better autonomous learning capacity, contrary to our expectation, especially in learning habit formation. Some studies have also shown that the effect of educational background on teachers’ learning capacity is not significant (Zhu and Jiang, 2021). During the interviews, teachers with doctoral degrees indicated that the mathematical professional learning they had conducted in scientific research was sufficient for their teaching practice and that there was little pressure for independent learning. It is noteworthy that the participants with doctoral degrees were not educated as professional teachers, they lacked teacher education backgrounds. The interviews also revealed that they paid more attention to the learning of mathematics professional theories, but not enough attention to the learning of mathematics teaching and learning theories, and lacked the awareness of mathematics curriculum standards, which made their knowledge in autonomous learning limited to mathematics professional knowledge rather than educational knowledge, and coupled with the high level of superiority brought by high education, these may lower their motivation for learning and development. Furthermore, according to the Education Statistics in T city, the number of teachers with doctoral degrees in mathematics and teacher training backgrounds is very small, which limits the number of people surveyed, and the results may change in the future as the cultivation of doctoral degrees in education in T city deepens, which is the focus of our further in-depth research in the future. In addition, the results revealed that the utilitarian tendency of teacher rewards and evaluation policies can hinder the improvement of teachers’ autonomous learning capacity. For example, in the interviews, teachers with doctoral degrees indicated that they have certain advantages in teaching in primary and secondary schools and evaluating teacher job titles in China and that they do not have to continue to do research or spend a lot of time studying for their job title rank after teaching. Therefore, they are more comfortable with their work status and less enthusiastic about learning. This is consistent with prior findings. For example, some studies have suggested that China’s teacher evaluation policies should focus on external static indicators such as educational background and job title (Wang and Si, 2011), without considering indicators that reflect the true quality of teachers, leading to a superficial quality evaluation framework (Wang, 2019).

In terms of job titles, there are no differences in the overall autonomous learning capacity among teacher groups. However, in learning habit formation, those with senior job titles do not demonstrate the expected advantages and even underperform those with intermediate job titles. The finding is consistent with existing research conclusions. For example, a previous study found that the learning motivation level of senior job title teachers is the lowest, and the difference with non-senior title teachers is significant (Liu and Liu, 2019). The reason could be because some teachers have become less motivated to work after promotion to senior titles or have moved to non-teaching positions, where they occupy senior title positions but do not teach (Wang and Wu, 2019). Moreover, interviews revealed that job title evaluations focus on exogenous indicators, while teachers at the senior level have reached the culmination of professional development with little hope of further promotion, which can reduce the willingness of some teachers with senior titles to seek autonomous learning and development.

In summary, the findings about differences in the autonomous learning capacity of mathematics teacher groups across demographic and contextual factors can contribute to the literature in this area. The implications are that measures need to be taken to enhance the autonomous learning capacity of particular teacher groups. For example, the unsatisfactory performance of teachers with senior titles and high levels of education may highlight the need for reforming job titles, pay scales, and teacher recruitment systems. For male teachers, those working at higher educational levels, and the middle-aged, targeted training and pedagogical research policies may be advisable.

### Future research suggestions

This study investigated the current state of teachers’ autonomous learning capacity, examined the differences across teacher groups, and discussed underlying reasons. First, the findings may inform future studies in developing more comprehensive scales, thus exploring factors affecting teachers’ autonomous learning capacity from a wider range of perspectives. Second, although this study has identified some significant influencing factors, their mechanisms of action still need further exploration. The relationship between correlated variables can be examined further to gain a clearer understanding of the paths through which teachers’ autonomous learning is affected. Finally, teacher policies have an important impact on the construction of the teaching team, the assessment is part of the efforts to provide evidence to inform teacher policies. A question to be addressed is whether artificial intelligence can be employed to conduct big data assessment of autonomous learning capacity comprehensively and in real-time to save effort.

## Data availability statement

The original contributions presented in the study are included in the article, further inquiries can be directed to the corresponding authors.

## Ethics statement

The studies involving human participants were reviewed and approved by Tianjin Normal University Academic Ethics Committee. The patients/participants provided their written informed consent to participate in this study.

## Author contributions

GW, YK, and XC contributed to the construction of concepts, evaluation index system, questionnaire preparation, introduction, and methodology. YZ and XC contributed to the literature review and theoretical background. YK and FL collected and analyzed the data. YK, YZ, and HH wrote the original draft of the manuscript. GW, YK, and YZ revised the manuscript. All authors contributed to the article and approved the submitted version.

## Funding

This study was supported by the key project of the National Social Science Foundation of China (Project No. AFA170008) and Major Project of “The Fourteenth Five-Year Plan of Teacher Education Development and the Future Development of Teacher Education” of China Higher Education Society’s Teacher Education Branch in 2020: Research on the Evaluation Index System and Assessment Model of Teachers’ Core Qualities and Abilities (Project No. 20ZSJSJYZG02).

## Conflict of interest

The authors declare that the research was conducted in the absence of any commercial or financial relationships that could be construed as a potential conflict of interest.

## Publisher’s note

All claims expressed in this article are solely those of the authors and do not necessarily represent those of their affiliated organizations, or those of the publisher, the editors and the reviewers. Any product that may be evaluated in this article, or claim that may be made by its manufacturer, is not guaranteed or endorsed by the publisher.
